# A deep learning model for predicting essential proteins based on an attention mechanism

**DOI:** 10.3389/fgene.2026.1688627

**Published:** 2026-04-15

**Authors:** Shunxian Zhou, Haodong Zhou, Sisi Chen, Yangtai Xu, Lei Wang

**Affiliations:** 1 College of Information Science and Engineering, Hunan Women’s University, Changsha, China; 2 College of Social Development, Hunan Women’s University, Changsha, China; 3 The First Hospital of Hunan University of Chinese Medicine, Changsha, China; 4 Intelligent Equipment School, Changsha Rail Transit Institute, Changsha, China; 5 Changsha Technology Innovation Center of Artificial Intelligence Large Model Training, Changsha University, Changsha, China

**Keywords:** deep learning model, essential proteins prediction, gene expression, orthology, subcellular localization

## Abstract

**Introduction:**

Essential proteins are key to cellular viability, yet their experimental identification is costly and time-consuming.

**Methods:**

In this study, DLAM is introduced as a deep learning framework that integrates four complementary biological cues, namely, domain composition, subcellular localization, orthology, and gene expression, together with a weighted protein–protein interaction network. The heterogeneous signals are encoded into compact representations and learned by an attention-enhanced network to score protein essentiality.

**Results:**

On the DIP dataset, DLAM achieves consistently better performance than representative centrality measures and conventional machine-learning classifiers. In a further expanded baseline study based on the larger BioGRID dataset containing more proteins, we conducted comparative experiments between DLAM and four recently proposed deep learning methods (TCBB2021, EPGAT, BMC2022, and ACDMBI). On the BioGRID dataset, we evaluated DLAM using stratified five-fold cross-validation. Across folds, DLAM achieves consistently strong discrimination and ranking performance (reported as the mean ± std. for ROC-AUC and AP) and maintains a stable F1-score under a validation-selected decision threshold. This suggests that, under the same evaluation protocol, DLAM has strong ranking and discrimination capability. Moreover, it also exhibits good and stable performance on other metrics such as accuracy, precision, recall, and F-measure.

**Discussion:**

These results indicate that jointly modeling multi-source biological information with interaction topology yields more reliable essential-protein prediction under class imbalance.

## 1 Introduction

Essential proteins play an indispensable role in the survival of organisms and the growth of human cells. Identification of essential proteins can not only contribute to understanding the processes and functions of cells but also promote the development of disease research and the analysis of drug targets. Since traditional experimental methods such as high-throughput technology (HU et al., 2023) and RNA screening (Ren et al., 2021) are time-consuming and expensive, in recent years, increasing numbers of computational methods have been developed to identify essential proteins, which can be mainly divided into two categories, namely, the methods based on networks and the methods based on machine learning. On the basis of the centrality–lethality rule proposed by Jeon et al. (2001), a series of methods based on networks, including the degree centrality (DC)-based method (Hahn and Kern, 2005), the information centrality (IC)-based method (Karen and Marvin, 1989), the betweenness centrality(BC)-based method (Joy et al., 2005), the closeness centrality (CC)-based method (Wuchty and Stadler, 2003), the subgraph centrality (SC)-based method (Estrada and Rodriguez-Velazquez, 2005), the neighbor centrality (NC)-based method (Wang et al., 2012), and the eigenvector centrality (EC)-based method () (Bonacich, 1987), have been proposed successively. In addition, Li et al. (2011) designed a prediction model called LAC to discover essential proteins based on the connection structure between proteins and their neighbors in protein–protein interaction networks (PPI networks). Considering that topological characteristics might have an important influence on protein essentiality, Pan et al. (2015) introduced a topological potential-based method to detect essential proteins. However, PPI networks contain false negatives and false positives. Moreover, the network-based methods, whose ability to extract features is limited, cannot extract more complex features from the biological information of proteins and PPI networks. Thus, the predictive performance of these network-based methods is not satisfactory. In order to overcome these problems, machine learning-based methods and deep learning-based methods have been developed in recent years to identify essential proteins by integrating PPI networks with the biological features of proteins. As for deep learning based-methods, Weihua et al. (2023) introduced a deep contextual representation learning model for identifying essential proteins by integrating multisource protein features. Zeng et al. (2021) constructed a deep learning model to automatically extract various biological characteristics to infer essential proteins. Schapke et al. (2022) designed an identification model called EPGAT to infer essential proteins by adopting attention-based graph neural networks. Yi et al. (2022) proposed a multiple-biological-information framework combining node2vec representations and depth-wise separable convolution to achieve end-to-end prediction of essential proteins. Lu and Tian (2024) proposed the community-division and multi-source fusion model ACDMBI. As for machine learning-based methods, Gustafson et al. (2006) applied the naive Bayes classifiers to detect essential proteins using targeted genome sequencing and comparative analysis. Acencio and Lemke (2009) constructed a machine learning framework to predict essential proteins by implementing the decision tree classifier to exact biological features for proteins. Dai et al. (2021) proposed an ensemble learning model that integrates multi-view learning with some base classifiers to detect essential proteins.

Inspired by the deep learning-based methods mentioned above, in this article, we propose a novel prediction model called DLAM for estimating protein essentiality. In DLAM, a new weighted protein–domain network is first established by collecting known protein–domain associations and computing the interaction similarities between domains. Next, the sparse autoencoder is exploited to extract domain features from the weighted protein–domain network. Meanwhile, the subcellular localization feature is extracted from the relevant biological network, and the orthologous feature and gene expression feature are obtained from the dataset. Then, four one-dimensional convolutional neural networks are adopted to extract the features of these four types of feature vectors, respectively, and their outputs are integrated into a new multi-channel feature map. After that, a one-dimensional convolutional neural network with an attention module named the convolutional block attention module (CBAM) (Woo et al., 2018) is designed to detect features from the multi-channel feature map and classify proteins. Finally, to assess the identification performance of DLAM, we compare it with representative methods, and the experimental results indicate that DLAM achieves better predictive performance than all these competitive models.

## 2 Materials and methods

**FIGURE 1:**
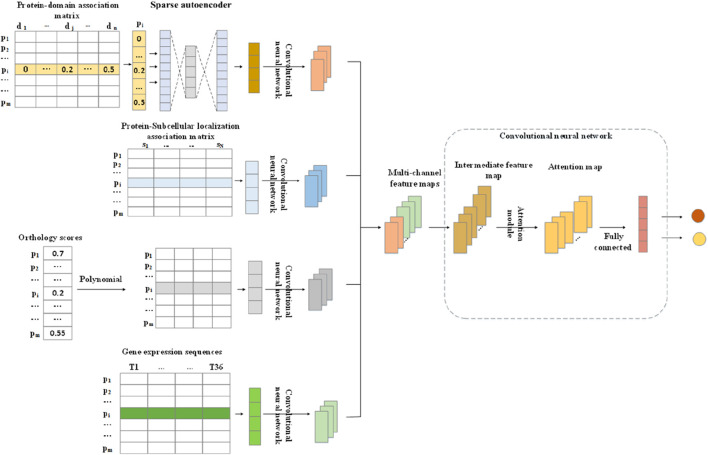
Flowchart of DLAM.

As illustrated in Figure 1, DLAM consists of two major parts. The first part is the feature extraction module, in which the domain feature vectors are extracted first from a newly constructed weighted protein–domain network by adopting the sparse autoencoder. Meanwhile, the subcellular localization feature vectors are extracted from the relevant biological network, gene expression sequences are collected from known gene expression data, and orthologous feature vectors are obtained through polynomial processing. Finally, four one-dimensional convolutional network modules are designed to learn the features of four types of feature vectors, in which the first two modules both consist of two convolutional layers with the Leaky ReLU activation function and one max pooling layer to learn the features of gene expression feature vectors and domain feature vectors, while the last two modules both consist of one convolutional layer with the Leaky ReLU activation function to extract the features of subcellular localization feature vectors and orthology feature vectors. Their outputs are integrated into a multi-channel feature map, which is regarded as the input to the second part. The second part consists of a convolutional layer, an attention layer, a max pooling layer, and three fully connected layers, which mainly extract the feature of the multi-channel feature map and classify proteins.

### 2.1 Extraction of the domain features

Evidence has demonstrated that protein essentiality is relevant to the domains (Peng et al., 2015). Hence, we choose the domain feature as one of the input features of the model. The extraction process is as follows. First, we download the domain dataset from the Pfam database (Bateman et al., 2003). For convenience, let Sp = {p1,p2, … ,pm} and Sd = {d1,d2, … ,dn} denote the sets of proteins and domains in the downloaded domain dataset, respectively. Then, we can construct a protein–domain network PDN = {Spd, Ipd}, in which Spd = Sp ∪ Sd and Ipd represents the set of edges between the proteins in Sp and the domains in Sd. Here, for any given protein pi and domain dj, if pi is associated with dj, we define that there is an edge between them in PDN. Based on the protein–domain network (PDN), we can further obtain an m×n dimensional protein–domain adjacency matrix, M_PDN_, according to the following Equation 1:

$$M_{PDN}\left(i,j\right)=\left\{\begin{array}{c} \;1\;if\;p_{i}\;associates\;with\;d_{j}\\ 0\;\;\;\;\;\;\;\;\;\;\;\;\;\;\;\;\;\;\;\;\;\;\;\;\;\;\;\;\;\;\;\;\;\;\;\;\;otherwise \end{array}\right..$$ (1)

Moreover, considering that similar domains may interact with similar proteins, based on above *M*_*PDN*_, for any two given domains *d*_*i*_ and *d*_*j*_, the Gaussian interaction profile kernel similarity between them can be calculated by the following Equations 2, 3:

$$SD\left(d_{i},d_{j}\right)=\exp\left(-\alpha_{d}\left\|ID\left(d_{i}\right)\right.-{\left.ID\left(d_{j}\right)\right\|}^{2}\right),$$ (2)

$$\alpha_{d}=\alpha_{d}^{\prime }/\left(\frac{1}{n}\sum_{k=1}^{n}{\left\|ID\left(d_{k}\right)\right\|}^{2}\right).$$ (3)

Here, *ID*(*d*_*i*_) denotes the *i*th column of *M*_*PDN*_. $\alpha_{d}$ is a parameter that adjusts the kernel bandwidth according to the new bandwidth parameter $\alpha_{d}^{\prime }$ .

Thereafter, by integrating the two matrices *M*_*PDN*_ and *SD*, a new m×n dimensional protein–domain matrix *M*_*WPDN*_ can be constructed by the following Equation 4:

$$M_{WPDN}\left(p_{i},d_{j}\right)=\frac{\max_{1\leq k\leq n}\left(M_{PDN}\left(p_{i},d_{k}\right)SD\left(d_{k},d_{j}\right)\right)+M_{PDN}\left(p_{i},d_{j}\right)}{2}.$$ (4)

Here, $M_{WPDN}\left(p_{i},d_{j}\right)$ denotes the associate score between the protein $p_{i}$ and the domain $d_{j}$ .

Because *M*_*WPDN*_ is a high-dimensional matrix, we adopt the sparse autoencoder (Hinton and Salakhutdinov, 2006) to extract low-dimensional domain feature vectors from the matrix *M*_*WPDN*_. An autoencoder, as an unsupervised learning method, can extract compressed features from a high-dimensional space effectively, which has been widely used in diverse fields such as image classification (Geng et al., 2017; Kemker and Kanan, 2017; Li et al., 2017) and video anomaly detection (Sabokrou et al., 2016; Dan et al., 2017; Sabokrou et al., 2017). An autoencoder involves two components, namely, the encoder and the decoder, whose structures are symmetrical. The number of hidden layers in the encoder is the same as that in the decoder. In this section, we construct a sparse autoencoder consisting of three layers, namely, the input layer with *n* input units, the hidden layer with *f* hidden units (1 < *f* < *n*), and the output layer with n neural units. Each row of *M*_*WPDN*_ is first treated as the input vector of the input layer and then fed into the hidden layer. The outputs of hidden layers are low-dimensional representations for proteins. The output layer restructures the outputs of the hidden layer. The encoder process and decoder process can be, respectively, expressed as the following Equations 5, 6:

$$O_{e}=\delta \left(W_{ih}v+b_{ih}\right),$$ (5)

$$O_{d}=\delta \left(W_{ho}O_{h}+b_{ho}\right).$$ (6)

Here, $W_{ih}$ is a f×n dimensional matrix, which represents weights of connections between the input layer and the hidden layer. *v* is the n-dimensional input vector (any row of matrix $M_{WPDN}$ ), and $b_{ih}$ is the bias term. $W_{ho}$ , a n×f dimensional matrix, denotes the weights of connections between the hidden and output layers. $b_{ho}$ is the bias term. δ denotes the activation function for the hidden and output layers, which will be set to the Satlin activation function and is represented as the following Equation 7:

$$St\left(x\right)=\delta \left(x\right)=\left\{\begin{array}{c} \;0\;\;if\;x<0\\ x\;if\;0\leq x\leq 1\;\\ \;1\;\;if\;x>1 \end{array}\right..$$ (7)

Through the back propagation, the parameters of the network are constantly updated. Finally, we use the trained parameters to compute the feature matrix, and the process of calculation is as the following Equation 8:

$$V_{dom}=\delta \left(W_{Tp}M_{WPDN}^{T}+b_{Tp}\right).$$ (8)

Here, $W_{Tp}$ is the trained connection weight between the input and hidden layers, which is a f×n dimensional matrix. $b_{Tp}$ is the trained bias term. The term $V_{dom}$ is the final domain feature matrix, in which each column represents the domain feature vector of the corresponding protein.

### 2.2 Extraction of protein subcellular features

In this section, we extract the subcellular localization feature vectors based on the subcellular localization information downloaded from the COMPARTMENTS database (Binder et al., 2014). For convenience, let $S_{sl}=\left\{s_{1},s_{2},.....,s_{N}\right\}$ and $S_{p}=\left\{p_{1},p_{2},....,p_{m}\right\}$ represent the sets of subcellular localizations and proteins in the downloaded dataset, respectively. Then, we construct a subcellular localization–protein association network $SPN=\left\{S_{sp},I_{sp}\right\}$ . Let $S_{sp}=S_{sl}\cup S_{p}$ and $I_{sp}=\left\{ed\left(p,s\right)\left|s\in \right.S_{sl},p\in S_{p}\right\}$ be the set of edges between the nodes in $S_{sl}$ and the nodes in $S_{p}$ . Moreover, for any given subcellular localization $s_{i}$ in $S_{sl}$ , let $NSP\left(s_{i}\right)=\left\{q|ed\left(q,s_{i}\right)\in I_{sp}\right\}$ denote the set of proteins that associate with $s_{i}$ ; then, the score of $s_{i}$ is obtained by the following Equation 9:

$$SC\left(s_{i}\right)=\left|NSP\left(s_{i}\right)\right|.$$ (9)

Here, $\left|NSP\left(s_{i}\right)\right|$ denotes the number of proteins associated with $s_{i}$ .

Thereafter, based on the scores of subcellular localizations, an N×m dimensional protein–subcellular association matrix $M_{ps}$ can be constructed by the following Equation 10:

$$M_{ps}\left(s_{i},p_{j}\right)=\left\{\begin{array}{c} \left.\;\right|NSP\left(s_{i}\right)|\;if\;s_{i}\;associates\;with\;protein\;p_{j}\\ \;0\;\;\;\;\;\;\;\;\;\;\;\;\;\;\;\;\;\;\;\;\;\;\;\;\;\;\;\;\;\;\;\;\;\;\;\;\;\;\;\;\;\;\;\;\;\;\;\;\;\;\;\;\;otherwise \end{array}\right..$$ (10)

Each column of the protein–subcellular localization association matrix $M_{ps}$ is the subcellular localization feature extracted for each protein.

### 2.3 Extraction of protein orthologous features

As one of the functional features of proteins, the information regarding orthology is highly correlated with the necessity of proteins. Thus, the orthology feature is also selected as an input feature of our model in this section. For adapting to the subsequent convolution operation, the dimension of the orthology data is increased using polynomials as follows: for any given protein $p_{i}$ , let $SO\left(p_{i}\right)$ denote its orthology score. Then, using the polynomial, $SO\left(p_{i}\right)$ is converted into a *t*-dimensional vector $V_{or}\left(p_{i}\right)=<{\left(SO\left(p_{i}\right)\right)}^{1},{\left(SO\left(p_{i}\right)\right)}^{2},...\;{\left(SO\left(p_{i}\right)\right)}^{t}>$ .

### 2.4 Integration of features

Through the above feature-extraction steps, we obtain four types of feature vectors, namely, the domain feature vector, the subcellular localization feature vector, the orthology feature vector, and the gene expression feature vector, which can be regarded as four different feature sequences to represent a protein in the PPI network. For convenience, for any given protein $p_{i}$ , let $\text{SG}\left(p_{i}\right)$ represent its gene expression feature vector with a dimension of 36, $V_{dom}\left(p_{i}\right)$ represent its domain feature vector learned by the sparse autoencoder, $V_{or}\left(p_{i}\right)$ denote its orthology features vector processed by the polynomial, and $V_{su}\left(p_{i}\right)$ be its subcellular localization feature vector. $V_{su}\left(p_{i}\right)$ is the *i*th column of $M_{ps}$ . Then, based on these four feature vectors, we further establish four convolutional neural networks to learn these feature vectors, respectively. Each convolutional neural network contains convolutional and activation layers. Although the ReLU activation function can solve the problem of vanishing gradients, it hinders the training process because the weights and biases are not updated when the input value to the ReLU function is less than 0. Hence, we finally choose the Leaky ReLU function as the activation function because it sets a parameter α to make the output value greater than 0 when the input value is less than 0.

As described above, these four convolutional neural networks generate four types of feature maps, respectively. For simplicity, let $F_{Gep}\in R^{1\times C_{1}\times L}$ , $F_{dom}\in R^{1\times C_{2}\times L}$ , $F_{sub}\in R^{1\times C_{3}\times L}$ , and $F_{ort}\in R^{1\times C_{4}\times L}$ denote these four types of feature maps, respectively. Here, $C_{1,}C_{2},C_{3},\text{and }C_{4}$ represent the channel numbers, and L stands for the length of feature maps. We concatenate these four maps into a new multi-channel feature map $F_{new}$ with $\left(C_{1}+C_{2}+C_{3}+C_{4}\right)$ channels according to the following Equation 11:

$$F_{new}=\left[F_{Gep},F_{dom},F_{sub},F_{ort}\right].$$ (11)

### 2.5 Implementation of the attention module

In this section, based on the newly obtained multi-channel feature map $F_{new}$ , an improved convolutional neural network module is designed to identify essential proteins, which comprise convolutional layers, activation layers, attention layers, and fully connected layers. The purpose of designing the attention layer is to extract effective features from the intermediate feature map learned by the convolutional layer. We adopt the CBAM, a lightweight module, to evaluate the importance of different features by sequentially calculating the attention map in both the channel and spatial dimensions. Before the attention layer, we set a batch normalization layer and a convolutional layer. The function of the batch normalization layer is to maintain the consistency of data distribution. Let $I_{d}\in \left(N,C_{1}+C_{2}+C_{3}+C_{4},L\right)$ denote N features maps $F_{new}\in \left(1,C_{1}+C_{2}+C_{3}+C_{4},L\right)$ . Here, N represents the batch size. The calculation process of batch normalization is described as the following Equation 12:

$$f_{b}=bn\left(I_{d}\right)=\frac{\gamma }{\sqrt{Var\left(I_{d}\right)+\epsilon }}*I_{d}+\left(\beta -\frac{\gamma E\left[I_{d}\right]}{\sqrt{Var\left(I_{d}\right)+\epsilon }}\right).$$ (12)

Here, $E\left[.\right]$ is the mean calculation, and $Var\left[.\right]$ is the variance calculation. γ and β are the learnable parameters. ϵ is a number that averts the denominator from becoming 0.

**FIGURE 2:**
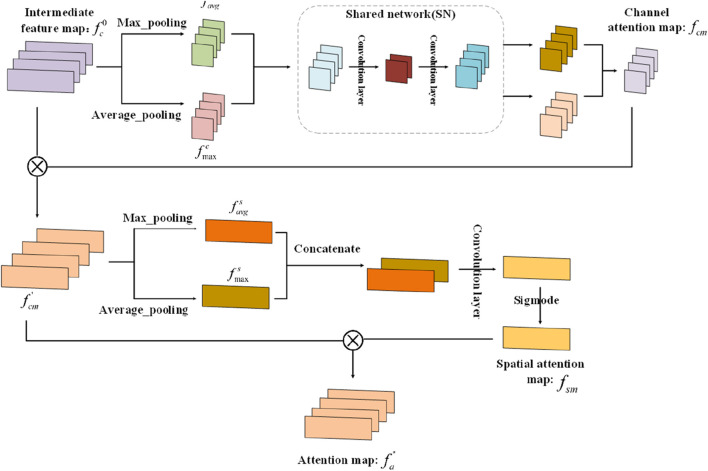
Operation process of the attention module.

Thereafter, we use the convolutional layer to learn the feature of $f_{b}$ , generating the feature map $f_{c}\in R^{N\times C\times L_{1}}$ . We apply the CBAM to compute the attention maps of $f_{c}\in R^{N\times C\times L_{1}}$ . Figure 2 shows the operation process of the attention module. Given a feature map $f_{c}^{0}\in R^{C\times L_{1}}$ from $f_{c}$ , its attention map is computed as follows: to obtain its channel attention map, the spatial dimension of the feature map $f_{c}^{0}\in R^{C\times L_{1}}$ is first compressed by exploiting average pooling and max pooling. The two types of features that are generated, respectively, are $f_{avg}^{c}\in R^{C\times 1}$ obtained using the average pooling operation and $f_{\max}^{c}\in R^{C\times 1}$ obtained using the max-pooling operation, as the following Equations 13, 14:

$$f_{avg}^{c}=AP\left(f_{c}^{0}\right),$$ (13)

$$f_{\max}^{c}=MP\left(f_{c}^{0}\right).$$ (14)

Here, AP(.) and MP(.) indicate the average and max pooling operations, respectively.

After that, according to the following Equations 15, 16, $f_{avg}^{c}$ and $f_{\max}^{c}$ are fed into a shared network SN that contains two convolutional layers. We choose the ReLU function as the activation function of the first convolutional layer.

$$f_{ap}=SN\left(f_{avg}^{c}\right),$$ (15)

$$f_{mp}=SN\left(f_{\max}^{c}\right).$$ (16)

The output features $f_{ap}$ and $f_{mp}$ of the shared network are integrated into a channel attention map, where the integration process is computed by the following Equations 17, 18:

$$f_{cm}=\varphi \left(f_{ap}+f_{mp}\right),$$ (17)

$$\varphi \left(x\right)=Sigmode\left(x\right)=\frac{1}{1+e^{-x}}.$$ (18)

Here, $\varphi \left[.\right]$ is the sigmoid activation function.

After calculating the channel attention map, the spatial channel map is computed by combining the input feature map $f_{c}^{0}$ with the channel attention map $f_{cm}$ as follows: first, the refined channel attention map $f_{cm}^{\prime }$ is calculated by integrating the channel attention map $f_{cm}$ with the input feature map $f_{c}^{0}$ as the following Equation 19:

$$f_{cm}^{\prime }=f_{cm}⊗f_{c}^{0}.$$ (19)

Next, the channel information is compressed by exploiting average pooling and max pooling separately. The two generated feature maps with one channel are computed by the following Equations 20, 21 separately:

$$f_{avg}^{s}={AP}_{s}\left(f_{cm}^{\prime }\right),$$ (20)

$$f_{\max}^{s}={MP}_{s}\left(f_{cm}^{\prime }\right).$$ (21)

${AP}_{s}$ and ${MP}_{s}$ are the average pooling operation and max pooling operation, respectively.

Furthermore, we can concatenate these two feature maps and input them to a convolutional layer. The produced spatial attention feature map $f_{sm}$ is calculated by the following Equation 22:

$$f_{sm}=\varphi \left({Cv}_{2}\left(\left[f_{avg}^{s};f_{\max}^{s}\right]\right)\right).$$ (22)

Here, ${Cv}_{2}\left[.\right]$ denotes the convolution operation. φ represents the sigmoid activation function.

Finally, the final attention feature $f_{a}^{\prime \prime }$ is computed by the fusion of the refined channel feature map $f_{cm}^{\prime }$ and the spatial attention feature map $f_{sm}$ as the following Equation 23:

$$f_{a}^{\prime \prime }=f_{cm}^{\prime }⊗f_{sm}.$$ (23)

### 2.6 Structure of DLAM

**FIGURE 3:**
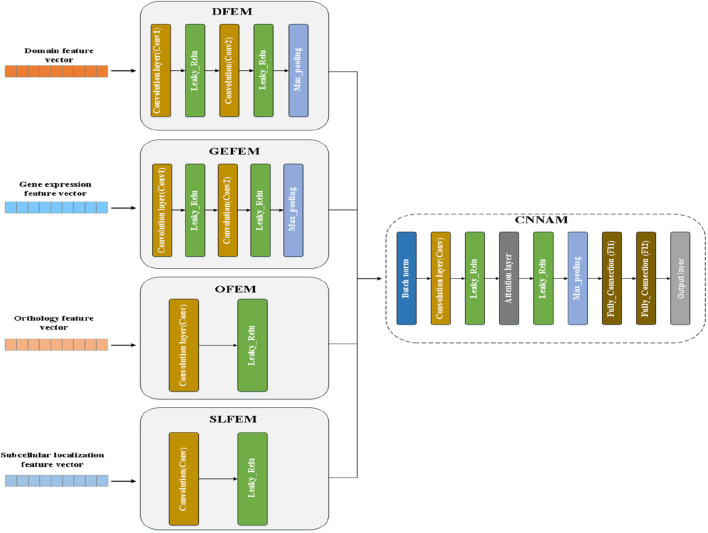
Structure of DLAM.

As indicated in Figure 3, DLAM mainly includes five components, namely, the domain feature extraction module (DFEM), the gene expression feature extraction module (GEFEM), the orthology feature extraction module (OFEM), the subcellular localization feature extraction module (SLFEM), and the convolutional neural network with the attention module (CNNAM). The workflow of DLAM is such that four modules, namely, DFEM, GEFEM, OFEM, and SLFEM, are first established to extract four different types of biological feature vectors for proteins; their outputs are integrated into multi-channel feature maps, and the multi-channel feature maps are input to the CNNAM module to predict essential proteins. The parameters of each layer of different modules are displayed in Table 1.

**TABLE 1:** Hyperparameters in DLAM.

Module name	Layer	Number of convolution kernels	Kernel size	Padding	Stride	Activation function	Nodes	Dropout
DFEM	Conv1	6	5	—	1	Leaky ReLU	—	—
	Conv2	10	4	—	2	Leaky ReLU	—	—
GEFEM	Conv1	6	5	—	1	Leaky ReLU	—	—
	Conv2	10	4	—	2	Leaky ReLU	—	—
OFEM	Conv	20	1	—	1	Leaky ReLU	—	—
SLFEM	Conv	20	1	—	1	Leaky ReLU	—	—
CNNAM	Conv	30	2	—	2	Leaky ReLU	—	—
	Fl1	—	—	—	—	Leaky ReLU	75	0.3
	Fl2	—	—	—	—	Leaky ReLU	35	—
	Output layer	—	—	—	—	—	2	—

### 2.7 Model training of DLAM

We used the MATLAB tool to extract and process three types of protein characteristics, namely, subcellular localization features, orthology features, and domain features, which have wide applicability in calculating the matrices and constructing large networks. To extract the domain features of proteins, we apply a sparse autoencoder to learn the representation vectors of domain features, where the sparse autoencoder consists of three different layers, and the hidden layer contains 36 neural units with the Satlin activation function. The values of the L2 regularization term and sparsity regularization are set to 0.01 and 4, respectively, while training the sparse autoencoder. Moreover, we use the polynomial to increase the dimension of the orthology feature vector to 12 while processing the orthology feature. After that, based on the feature vectors extracted by MATLAB, PyTorch is utilized to establish the deep learning framework to learn the information of protein sequences and classify proteins; in addition, the following cross entropy function is chosen as the loss function:

$$EF\left(x\right)=-\sum_{x}l_{x}\log p_{x}.$$ (24)

In above Equation 24, l denotes the true label of the protein, while p represents the predicted label.

Furthermore, we adopt the Adam optimizer and set the learning rate to 0.009 when training DLAM. In addition, the batch size is set to 20. Because continuous training may lead to overfitting, the early stopping method is adopted to determine whether the model should stop training according to its performance on the validation dataset. The condition of stopping training is that the loss of the validation dataset does not decrease within 10 epochs.

### 2.8 Collection of datasets

In comparative experiments between DLAM and centrality-based methods and traditional machine-learning methods, six different datasets were used, namely, the PPI dataset, the essential-protein dataset, the domain dataset, the gene expression dataset, the subcellular localization dataset, and the orthology dataset. Specifically, the PPI dataset was downloaded from the DIP database (Xenarios et al., 2002); after removing duplicate interactions and self-interactions, it contained 5,093 proteins and 24,743 interactions. The essential-protein dataset comprised 1,293 essential proteins collected from four databases, namely, SGDP (Wilson and Roach, 2012), DEG (Ren and Lin, 2009), SGD (Cherry et al., 1998), and MIPS (Mewes et al., 2006). By matching the downloaded essential-protein dataset with the PPI dataset, 1,167 essential proteins were finally retained. The protein–domain dataset was obtained from the Pfam database (Peng et al., 2015) and contained 1,081 domains after processing. Based on the newly downloaded PPI and domain datasets, we constructed a protein–domain matrix of size 5,093 × 1,081. In addition, the gene expression dataset was collected from the GEO database (Tu and et al., 2005), containing the expression profiles of 6,776 genes across 36 samples, while the orthology dataset was gathered from the InParanoid database (Ostlund et al., 2010). Finally, the subcellular localization dataset was downloaded from the COMPARTMENTS database (Binder et al., 2014), from which 11 subcellular localization categories closely related to essential proteins—such as endoplasmic, cytoskeleton, and Golgi bodies—were selected.

In further comparative experiments between DLAM and four major deep learning methods proposed in recent years, the BioGRID dataset with a larger number of proteins was adopted. The PPI data were downloaded from BioGRID (Table 3), and only the interaction data of yeast (*Saccharomyces cerevisiae*) were retained. After removing duplicate edges and self-loops, we obtained 4,326 proteins and 24,691 interactions. The essential-protein set was still collected from SGDP, DEG, SGD, and MIPS; by considering the intersection with the BioGRID protein set, 1,124 essential proteins were obtained for labeling.

For missing data handling, multi-source protein annotations are often incomplete in practice. To make our pre-processing reproducible and reduce ambiguity, we handle missing modalities using an explicit, fold-consistent procedure consisting of the following steps: (1) identifier harmonization: we map proteins across sources using stable identifiers (BioGRID systematic names when available; otherwise UniProt/SGD identifiers). Proteins are treated as the same entity only when a one-to-one mapping is available; ambiguous mappings are excluded. (2) Modality-specific coverage: for each modality (gene expression, orthology, domain, and subcellular localization), we compute a feature vector for every protein where possible. We record a binary availability flag for each modality of each protein. (3) Imputation with missingness flags: if a protein lacks a modality, we set that modality’s feature vector to all zeros and concatenate the corresponding availability flag(s) to the input. This makes the missingness explicit to the model rather than implicitly treating missing values as informative numeric values. (4) Fold-aware normalization: within each CV fold, we fit feature normalization parameters (mean/standard deviation) on the training split only and apply the same transformation to the validation and test splits. This prevents information leakage. (5) Sensitivity check (recommended): we optionally repeat evaluation after excluding proteins that are missing a major modality (e.g., expression) to verify that the conclusions are not driven by coverage artifacts.

## 3 Results and analysis

### 3.1 Evaluation metrics

To evaluate the predictive performance and generalization ability of DLAM, this section adopts five evaluation metrics, namely, accuracy, precision, recall, F-measure, and the area under the curve (AUC). Among them, accuracy is calculated as shown in Formula 25, which denotes the ratio of true positive samples and true negative samples to all samples in the testing dataset. Precision is the proportion of true positive samples among samples that are predicted to be positive in the testing dataset, which can be calculated according to Formula 26. Recall is the proportion of true positive samples among the true positive samples in the testing dataset, which can be calculated according to Formula 27. Thereafter, based on the concepts of precision and recall, F-measure can be defined according to Formula 28.

$$Accuracy=\frac{\left(TP+TN\right)}{\left(TP+TN+FP+FN\right)},$$ (25)

$$Precision=\frac{TP}{\left(TP+FP\right)},$$ (26)

$$Recall=\frac{TP}{\left(TP+FN\right)},$$ (27)

$$F-measure=\frac{2*precision*recall}{precision+recall}.$$ (28)

Here, TP, FP, TN, and FN are the numbers of true positive samples, false positive samples, true negative samples, and false negative samples, respectively. The AUC, area enclosed by the receiver operating characteristics (ROC) curve and the coordinate axis, is a performance metric that reflects the predictive ability of the classifier. Since the ROC curve is generally above the line *y = x*, it means that the value of AUC ranges from 0.5 to 1. Hence, it shows that the learning ability of the model improves as the AUC approaches 1.

Essential-protein prediction is intrinsically imbalanced on both DIP2010 and BioGRID (positives are a minority). In such settings, ROC-AUC alone can overstate the performance because it is less sensitive to changes in precision at low recall. We therefore report precision–recall (PR) curves and average precision (AP) as primary ranking metrics, together with F1-score computed at a fold-specific threshold selected on the validation set. This combination provides complementary views, where ROC-AUC reflects the overall separability, AP summarizes the ranking quality under imbalance, and F1 captures the operating-point performance.

### 3.2 Comparison with centrality-based measures

To accurately assess the predictive performance of DLAM, in this section, we compare it with eight centrality-based measures, namely, DC, IC, EC, CC, NC, CoEWC, ION, and TEGS. While implementing these methods, we adopt the following three steps to calculate their assessment metrics. First, we calculate the scores of proteins by reproducing these centrality-based methods.

**FIGURE 4:**
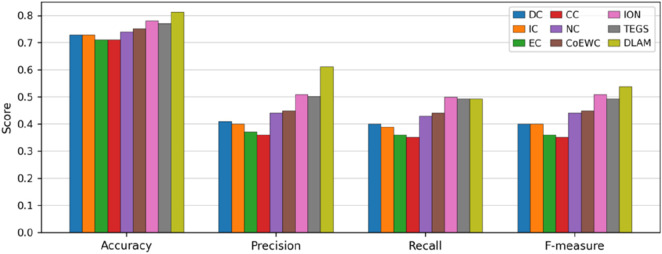
Comparison of the performances of DLAM with that of the centrality methods, including DC, CC, ION, IC, NV, TEGS, EC, and CoEWC.

**FIGURE 5:**
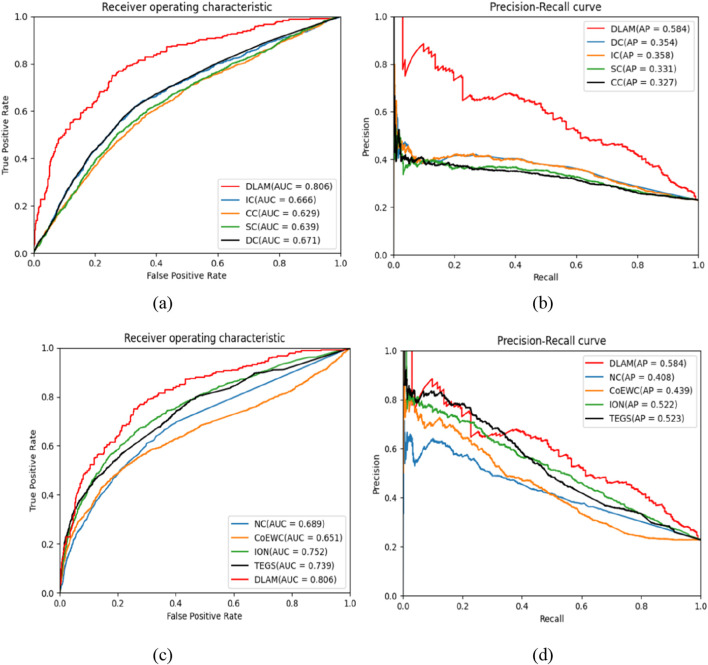
Comparison of the ROC and PR curves of DLAM with those of the centrality methods, where part **(a)** includes the comparison of ROC curves between DLAM and IC, CC, SC, DC, part **(b)** includes the comparison of PR curves between DLAM and IC, CC, SC, DC, part **(c)** includes the comparison of ROC curves between DLAM and NC, CoeWC, ION, TEGS, and part **(d)** includes the comparison of PR curves between DLAM and NC, CoeWC, ION, TEGS.

Second, by sorting the scores of proteins in descending order, we specify the top 1,143 proteins as candidate essential proteins and the remaining 3,950 proteins as non-essential proteins. Finally, five evaluation metrics, namely, accuracy, precision, recall, F-measure, and AUC of each method, are computed successively, and comparison results are shown in Figures 4, 5, respectively. As depicted in Figure 4, it can be observed that the predictive performance of DLAM exceeds that of these centrality-based competitive methods.

Moreover, Figures 5a,c show that the AUC of DLAM is higher than that of these centrality-based models, and the ROC curve of DLAM is also higher than that of the centrality-based models. In addition, in Figure 5b, the location of the PR curve is the highest when comparing it with the PR curves of all the centrality-based models. We note that the TEGS method performs better than the other centrality methods. In Figure 5d, the PR curve of DLAM overlaps the PR curve of TEGS slightly in the range of recall from 0.05 to 0.35, but the AP score of DLAM is 0.061 higher than that of TEGS. Hence, we can conclude that DLAM can achieve better predictive effects than the centrality-based methods.

### 3.3 Comparison with machine learning-based methods

**FIGURE 6:**
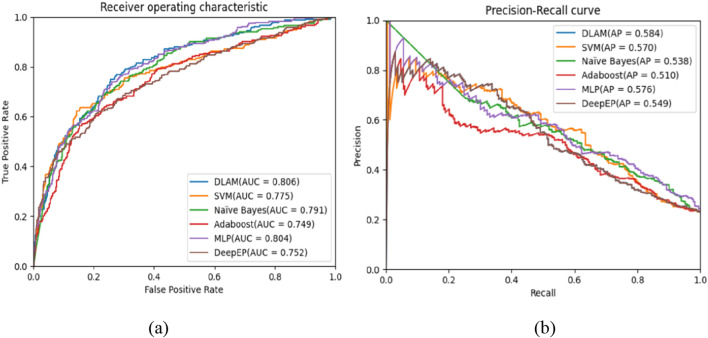
Comparison of the ROC and PR curves of the DLAM with machine learning-based methods, where part **(a)** includes the comparison of the ROC curves between DLAM and five competing machine learning-based methods, and part **(b)** includes the comparison of the PR curves between DLAM and these five competing machine learning-based methods.

In recent years, several machine learning measures have been introduced to infer essential proteins. In this section, we select five models, namely, SVM, AdaBoost, naïve Bayes, MLP, and DeepEP, to compare their predictive performances with that of DLAM. The SVM, AdaBoost, and naïve Bayes are shallow machine learning-based methods, and we utilize the scikit-learn library in Python to implement them. During simulation, all training parameters are set to the default value. As a new branch of the machine learning field, DeepEP has a powerful ability to learn and obtain more feature information. During experiments, the PPI dataset obtained from DIP database is divided into the training and testing datasets. The training dataset makes up 80% of the raw dataset and contains 934 essential proteins and 3,140 non-essential proteins. The remaining data are regarded as the testing dataset, consisting of 233 essential proteins and 786 non-essential proteins. The proportion of essential and non-essential proteins is consistent with that of the raw dataset, and the training dataset is input into each predictive model to train the model parameters. The testing dataset is adopted to test the performance of each model. Additionally, for the sake of fairness, each machine learning method keeps the same input, and the resulting values of the assessment metrics achieved by DLAM and these competitive methods are shown in Table 2. Table 2 shows that the AUC of DLAM is much higher than that of SVM (0.775), MLP (0.806), naïve Bayes (0.791), AdaBoost (0.749), and DeepEP (0.752) simultaneously. Figure 6 shows the ROC and PR curves of DLAM and five types of methods, from which it can be observed that although the ROC curves of the naïve Bayes and DLAM overlap, the AUC value achieved by DLAM is 0.806, which is better than that of the naïve Bayes method.

**TABLE 2:** Performance comparison between DLAM and machine learning-based methods.

Method	Accuracy	Precision	Recall	F-measure	AUC
SVM	0.819	0.699	0.369	0.483	0.775
MLP	0.806	0.621	0.386	0.476	0.804
Naïve Bayes	0.797	0.556	0.554	0.555	0.791
AdaBoost	0.789	0.558	0.369	0.444	0.749
DeepEP	0.759	0.479	0.577	0.523	0.752
DLAM	0.811	0.605	0.494	0.544	0.806

### 3.4 Comparison with deep learning-based methods

To make the comparison more comprehensive, we additionally evaluate our method against four recently proposed deep learning-based methods (baseline methods) for essential protein prediction, namely, a multi-source biological information integration framework (TCBB2021) (Zeng et al., 2021), the graph-attention-based EPGAT model (TCBB2022) (Schapke et al., 2022), a multiple-biological-information framework combining node2vec representations and depth-wise separable convolution (BMC2022) (Yi et al., 2022), and the community-division and multi-source fusion model (ACDMBI2024) (Lu and Tian, 2024).

A fair baseline comparison requires clarity on both the input features and the implementation details used for each method. In our experiments, we follow each baseline’s published feature design when the required annotations are available in our curated datasets; when a baseline requires modalities that are not provided for a given benchmark, we evaluate that baseline under the maximal common feature subset shared by all the methods on that benchmark. This choice prevents any single method from accessing additional information that is unavailable to others.

In addition, we re-implement the baselines under a unified training and evaluation pipeline (same train/validation/test protocol within each CV fold, same early-stopping rule, and the same metric-driven threshold selection on the validation split). Some architectural or preprocessing details (e.g., the specific node embedding algorithm, feature normalization, or the original hyperparameter defaults) may, therefore, differ from those used in the original publications. Accordingly, the baseline results should be interpreted as a controlled comparison under consistent protocols rather than as a strict reproduction of each method’s best-reported configuration.

**FIGURE 7:**
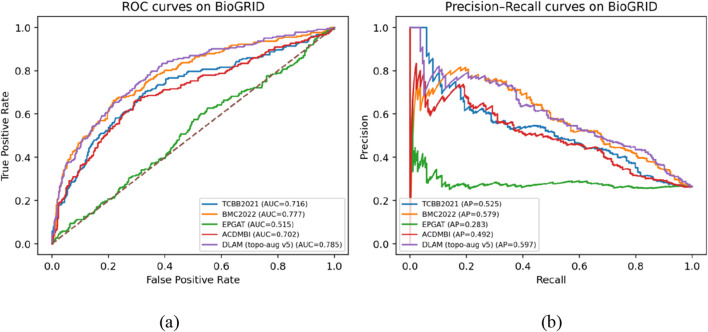
Comparison of the ROC and PR curves of the DLAM with recently proposed deep learning-based methods, where part **(a)** includes the comparison of ROC curves between DLAM and four competing deep learning-based methods, and part **(b)** includes the comparison of PR curves between DLAM and these four competing deep learning-based methods.

In the comparative experiments, we used the BioGRID dataset, which contains a larger number of proteins. The performance comparison between DLAM and the recently proposed deep learning-based methods is shown in Table 3, and the comparison of the ROC and PR curves of DLAM with the recently proposed deep learning-based methods is shown in Figure 7.

**TABLE 3:** Performance comparison between DLAM and deep learning-based methods.

Method	Accuracy	Precision	Recall	F-measure	AUC	AP
TCBB2021	0.671	0.426	0.709	0.532	0.716	0.525
BMC2022	0.751	0.523	0.601	0.559	0.777	0.579
EPGAT	0.292	0.267	0.964	0.418	0.515	0.283
ACDMBI	0.657	0.410	0.686	0.513	0.702	0.492
DLAM	0.720	0.479	0.700	0.568	0.785	0.597

The experimental results show that, overall, DLAM achieves higher AUC (0.785) and AP (0.597) than those of the other four deep learning methods. In particular, for the class-imbalanced task of essential protein identification, AP better reflects the ability to rank truly essential proteins near the top. This indicates that under the same evaluation protocol, DLAM demonstrates strong ranking and discrimination capability, and it also exhibits good and stable performance on the other metrics.

More specifically, among the four major deep learning approaches proposed in recent years, BMC2022 shows relatively strong overall performance. DLAM is ranked only slightly lower than BMC2022 in precision and accuracy, while outperforming BMC2022 on the remaining metrics—especially achieving better results on the threshold-independent AUC/AP—suggesting that DLAM is more suitable for scenarios where candidate essential proteins need to be prioritized by ranking. Compared with TCBB2021, DLAM achieves higher accuracy, precision, F-measure, AUC, and AP, with recall being only 0.009 lower, which is a very small trade-off. Compared with ACDMBI, DLAM performs better on all the core metrics. These results indicate that under a larger and noisier PPI setting, such as BioGRID, DLAM provides more stable discrimination and ranking performance and is superior to TCBB2021 and ACDMBI. Compared with EPGAT, the results are quite typical: EPGAT attains an extremely high recall (0.964), while the other major metrics are relatively low. This indicates that under the current setting, EPGAT labels a large portion of samples as essential proteins, leading to high recall but substantially more false positives; its ranking performance is also close to random (AUC ≈0.5). This may also be because under the combined effects of noisy edges and class imbalance in the BioGRID dataset, pure graph-attention-based message passing is more prone to over-smoothing/over-propagation and threshold bias, thereby leading to the “high recall but low precision and low discriminability” pattern.

Under the experimental scale in this study, DLAM’s computational cost can be regarded as “moderate for node-level deep models,” and it is clearly lower than that of EPGAT, which requires whole-graph message passing. Compared with TCBB2021/BMC2022, DLAM is slightly more computationally demanding, but it still maintains acceptable training and inference overhead. ACDMBI has training/inference costs that are close to those of an MLP, but it additionally involves a one-time community-detection preprocessing cost. The computational cost comparison is shown in Table 4.

**TABLE 4:** Computational cost comparison between DLAM and deep learning-based methods.

Method	Training time	Inference time	Peak memory	Notes
TCBB2021	Low	Low	Low	Node-wise MLP, *O(Nd)*
BMC2022	Low–medium	Low–medium	Low–medium	Node-wise fusion, small conv/attention
EPGAT	High	High	High	Graph attention message passing, *O(Ed)*
ACDMBI	Low–medium	Low–medium	Low–medium	+ One-time community detection
DLAM	Medium	Medium	Low–medium	Node-wise CNN + attention (short sequence)

### 3.5 Ablation study

**FIGURE 8:**
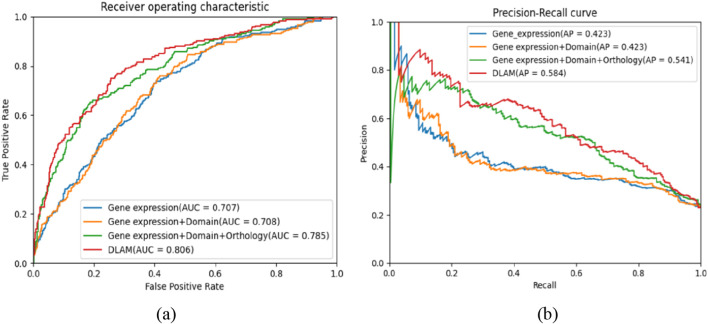
Comparison of the ROC and PR curves of the DLAM model in various feature combinations, where part **(a)** includes the comparison of ROC curves of DLAM in various combinations, and part **(b)** includes the comparison of PR curves of DLAM in various combinations.

Because inputs of DLAM are four types of feature vectors of proteins (gene expression feature vectors, domain feature vectors, orthology feature vectors, and subcellular localization feature vectors), for the sake of investigating each feature used by the DLAM model, we conduct ablation experiments according to different feature combinations. The gene expression feature vectors are first selected as the input of DLAM. According to the output of DLAM, the prediction performance of the DLAM model with only the gene expression feature vector is appraised. Afterward, the other feature vectors (domain feature vectors, orthology feature vectors, and subcellular localization feature vectors) are incrementally input into the DLAM model to analyze their impact on the evaluation indicators of DLAM. The experimental results are shown in Table 5. From Table 5, we observe that these protein features have a positive influence on the prediction ability of DLAM. Specifically, after adding the orthology feature, the model’s performance is significantly improved by combining gene expression feature vectors, domain feature vectors, and orthology feature vectors, with the AUC increasing from 0.707 to 0.785 and recall increased by 0.185. Therefore, the orthology information shows great potential for enhancing model performance. Notably, the AUC reaches the highest value of 0.806 when all feature vectors are included in the model. Consequently, it illustrates that these selected features are effective. Figure 8 shows the ROC and PR curves for different feature combinations. We observe that the AUC and AP scores of DLAM are 0.806 and 0.584, respectively, which are higher than those of DLAM with three other types of feature combinations. It can be concluded that the combination of all features achieves a better effect than other three types of combinations.

**TABLE 5:** Performance comparison of the DLAM model in different feature combinations.

Feature data	Accuracy	Precision	Recall	F-measure	AUC
Gene expression	0.776	0.547	0.124	0.203	0.707
+Domain	0.782	0.608	0.133	0.218	0.708
+Orthology	0.805	0.649	0.318	0.427	0.785
+Sub-localization (DLAM)	0.811	0.605	0.494	0.544	0.806

**FIGURE 9:**
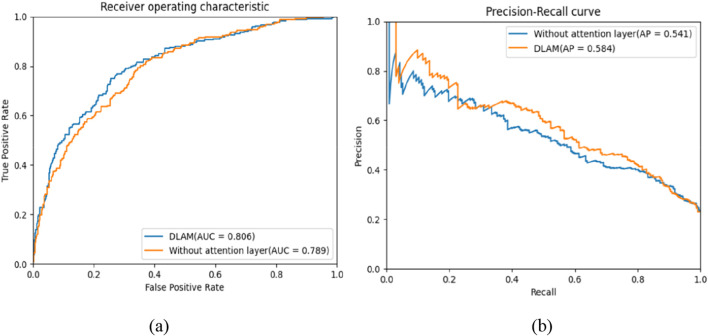
ROC and PR curves of the DLAM model and the DLAM model without the attention layer, where part **(a)** includes the comparison of ROC curves between the DLAM and the DLAM without the attention layer, and part **(b)** includes the comparison of PR curves between the DLAM and the DLAM without the attention layer.

To extract more features that are relevant to the importance of proteins, we add an attention layer to the DLAM model. To verify the effectiveness of the attention layer, we implement an ablation study for the attention layer. Table 6 shows the experimental results, which include evaluation indexes of the DLAM model without the attention layer and the DLAM model with the attention layer. It can be observed from Table 6 that the accuracy, precision, recall, and F-measure of the DLAM model with the attention layer are higher than those of the DLAM model without the attention layer. The ROC and PR curves are shown in Figure 9. From Figure 9, it can be observed that the AUC and AP of DLAM with the attention layer are 0.017 higher and 0.043 higher than those of the DLAM model without the attention layer. Thus, this indicates that the attention layer can enhance the performance of the model to a certain degree.

**TABLE 6:** Effect of the attention layer on the DLAM model.

Model	Accuracy	Precision	Recall	F-measure	AUC
Without attention layer	0.805	0.639	0.335	0.439	0.789
DLAM	0.811	0.605	0.494	0.544	0.806

To avoid overfitting to a single random split and ensure comparable tuning effort across methods, we select hyperparameters within each CV fold using the corresponding training/validation split only. For DLAM and each baseline, we perform a bounded search over the learning rate, weight decay, dropout, hidden dimension, number of layers (when applicable), and loss settings (e.g., class-weighted BCE or focal loss). The search is conducted either as a small grid or a randomized search under a fixed budget; all methods are allocated the same budget (the number of trials) and the same early-stopping rule. Within each fold, candidate models are trained on the training split and evaluated on the validation split. We select the configuration that maximizes the validation ROC-AUC as the primary criterion, with AP as the secondary tie-breaker. The decision threshold used for reporting F1/precision/recall is chosen on the validation split of the same fold. No information from the held-out test split is used for tuning.

The PPI dataset is unbalanced, with a ratio of essential to non-essential proteins is approximately 1:4. The training of the DLAM model is based on the raw dataset. To validate the reliability of DLAM’s performance on the raw dataset, we use random undersampling measure to assess the model’s predictive performance at different ratios. We create four datasets by splitting the raw dataset according to four different ratios, namely, 1:1, 1:1.5, 1:2, and 1:2.5, which are then used to train the DLAM model. Table 7 shows the comparison result among the models with different ratios. We can observe that the accuracy and AUC of DLAM trained with the raw dataset are 0.811 and 0.806, which are higher than those of the other three ratios, namely, 1:1 (0.726 and 0.778), 1:1.5 (0.717 and 0.791), 1:2 (0.765 and 0.805), and 1:2.5 (0.781 and 0.803). Overall, utilizing the raw dataset is more applicable to the DLAM model.

**TABLE 7:** Performance of DLAM with different ratios of essential proteins.

Ratio (Essential: non-essential)	Accuracy	Precision	Recall	F-measure	AUC
1:1	0.726	0.709	0.769	0.738	0.778
1:1.5	0.717	0.704	0.509	0.591	0.791
1:2	0.765	0.675	0.568	0.617	0.805
1:2.5	0.781	0.652	0.498	0.564	0.803
DLAM (raw dataset)	0.811	0.605	0.494	0.544	0.806

### 3.6 Cross-validation results

To mitigate potential bias introduced by a single random train/validation/test split and further assess the stability of DLAM, we conducted a stratified five-fold cross-validation on the BioGRID yeast dataset. In each fold, approximately 80% of the samples were used for model development, and the remaining 20% were held out for testing; within the development set, a validation subset was further separated to follow the same validation protocol as in our main experiments (i.e., the overall 60/20/20 ratio with validation-based threshold selection). Consistent with the BioGRID comparative experiments, the input representation was constructed by integrating gene expression features, orthology features, and BioGRID-derived PPI embeddings, with simple topology descriptors (log-degree and k-core index) included as auxiliary cues. Table 8 summarizes the fold-wise performance in terms of accuracy, precision, recall, F-measure, and ROC-AUC. Overall, DLAM shows stable performance across different folds, achieving accuracy = 0.681 ± 0.042, precision = 0.435 ± 0.034, recall = 0.713 ± 0.061, F-measure = 0.538 ± 0.025, and ROC-AUC = 0.756 ± 0.014 (mean ± standard deviation). For completeness, we also report AP = 0.531 ± 0.032. These results indicate that the observed performance is not driven by a particular data split and that DLAM maintains consistent predictive capability under different partitions.

**TABLE 8:** DLAM’s five-fold cross-validation (CV) results on BioGRID (with approximately 20% of the samples used as the test set in each fold).

Fold	Accuracy	Precision	Recall	F-measure	AUC	AP
1	0.714	0.466	0.698	0.559	0.756	0.544
2	0.680	0.426	0.679	0.523	0.755	0.537
3	0.703	0.457	0.756	0.570	0.779	0.564
4	0.699	0.446	0.640	0.526	0.747	0.479
5	0.609	0.380	0.791	0.513	0.744	0.533

### 3.7 Statistical significance testing

Following the “unified pipeline” setting adopted in our revised baseline comparison, all baselines were evaluated using the same data splits and a consistent feature construction pipeline; therefore, the fold-wise baseline scores should be interpreted as controlled comparisons under a standardized protocol rather than exact reproductions of each method’s original and potentially dataset-specific configuration.

For each metric (ROC-AUC, average precision (AP), and F1), we applied a paired t-test and a Wilcoxon signed-rank test on the per-fold scores (n = 5 paired observations). To control for multiple comparisons across the four baselines, we additionally report Holm-adjusted *p*-values for each metric. The cross-fold experimental results are shown in Table 9.

**TABLE 9:** Summary of the paired tests (two-sided) for DLAM *versus* each baseline.

Metric	Baseline	Mean (DLAM)	Mean (Baseline)	MeanDiff	p-t-test	P_Wilcoxon	p_w_holm
AUC	TCBB2021	0.756 ± 0.014	0.758 ± 0.015	−0.002	0.8354	0.8125	1.0000
AUC	BMC2022	0.756 ± 0.014	0.748 ± 0.024	+0.008	0.4220	0.6250	1.0000
AUC	EPGAT	0.756 ± 0.014	0.749 ± 0.048	+0.007	0.7730	1.0000	1.0000
AUC	ACDMBI	0.756 ± 0.014	0.750 ± 0.006	+0.006	0.2498	0.1875	0.7500
AP	TCBB2021	0.531 ± 0.032	0.521 ± 0.036	+0.010	0.5554	0.6250	1.0000
AP	BMC2022	0.531 ± 0.032	0.517 ± 0.034	+0.015	0.4745	0.6250	1.0000
AP	EPGAT	0.531 ± 0.032	0.525 ± 0.066	+0.007	0.8536	1.0000	1.0000
AP	ACDMBI	0.531 ± 0.032	0.550 ± 0.019	−0.018	0.2948	0.3125	1.0000
F1	TCBB2021	0.538 ± 0.025	0.541 ± 0.008	−0.003	0.8149	0.8125	1.0000
F1	BMC2022	0.538 ± 0.025	0.534 ± 0.030	+0.004	0.6219	0.8125	1.0000
F1	EPGAT	0.538 ± 0.025	0.540 ± 0.041	−0.002	0.9261	1.0000	1.0000
F1	ACDMBI	0.538 ± 0.025	0.515 ± 0.017	+0.023	0.0190	0.0625	0.2500

Overall, the paired tests indicate that the fold-to-fold differences are modest and, after correcting for multiple comparisons, do not reach conventional statistical significance at α = 0.05 for ROC-AUC or AP. This outcome is consistent with the limited sample size (five-fold), which constrains statistical power; nevertheless, reporting paired tests provides an explicit statistical check of the comparative claims.

For transparency, we provide the complete per-fold metric files and a reproducible script to run the paired t-tests and Wilcoxon tests.

## 4 Discussion and conclusion

In this article, a deep learning method named DLAM is developed to detect essential proteins. In DLAM, four types of biological features, namely, the domain feature, the subcellular feature, the orthology feature, and the gene expression feature, of proteins are selected as the input features of an improved deep learning model to infer potential essential proteins. In addition, in order to evaluate the predictive effects of DLAM, three types of representative measures, namely, the centrality-based measures, the machine learning-based methods, and the deep learning-based methods, are compared with DLAM in detail. The experimental results show that DLAM achieves competitive, though not statistically distinguishable, performance relative to the recent baselines.

## 5 Limitations

Although we extend the evaluation from DIP to the yeast BioGRID benchmark and additionally report stratified five-fold cross-validation, the empirical evidence is still limited to *S. cerevisiae* and the specific essentiality labels curated from available databases. The model’s performance may change for other organisms, alternative PPI repositories, or under different definitions of essentiality (e.g., condition-specific essential genes). Establishing broader external validity will require multi-species benchmarks and independent interaction sources with matched label curation.

DLAM also relies on multi-source biological annotations (gene expression, domain, orthology, and subcellular localization). In practice, these fields are heterogeneous across databases and often have missing data or are noisy for a subset of proteins. Such incompleteness can reduce effective coverage and may implicitly favor proteins with richer annotations, which is particularly relevant when transferring the pipeline to organisms with sparse functional metadata.

In addition, part of the input representation is derived from the observed interaction network (e.g., PPI embeddings and topology-derived descriptors). PPI networks are known to contain both false positives and false negatives, and the downstream representations may inherit these biases. We did not systematically quantify how sensitive the results are to alternative network preprocessing choices (e.g., confidence filtering and edge de-duplication rules) or to different embedding configurations, which may affect robustness on larger or noisier networks. The baseline results should be interpreted as a controlled comparison under consistent protocols rather than as a strict reproduction of each method’s best-reported configuration.

## Data Availability

The original contributions presented in the study are included in the article/Supplementary Material, further inquiries can be directed to the corresponding authors.
